# Whiplash injury

**DOI:** 10.34172/aim.2022.45

**Published:** 2022-04-01

**Authors:** Halil Keskin, Berhan Pirimoglu

**Affiliations:** ^1^Ataturk University Faculty of Medicine, Department of Pediatrics, Division of Pediatric Intensive Care Unit, 25240 Erzurum, Turkey; ^2^Ataturk University Faculty of Medicine, Department of Radiology, Division of Pediatric Radiology, 25240 Erzurum, Turkey

**Figure 1:**
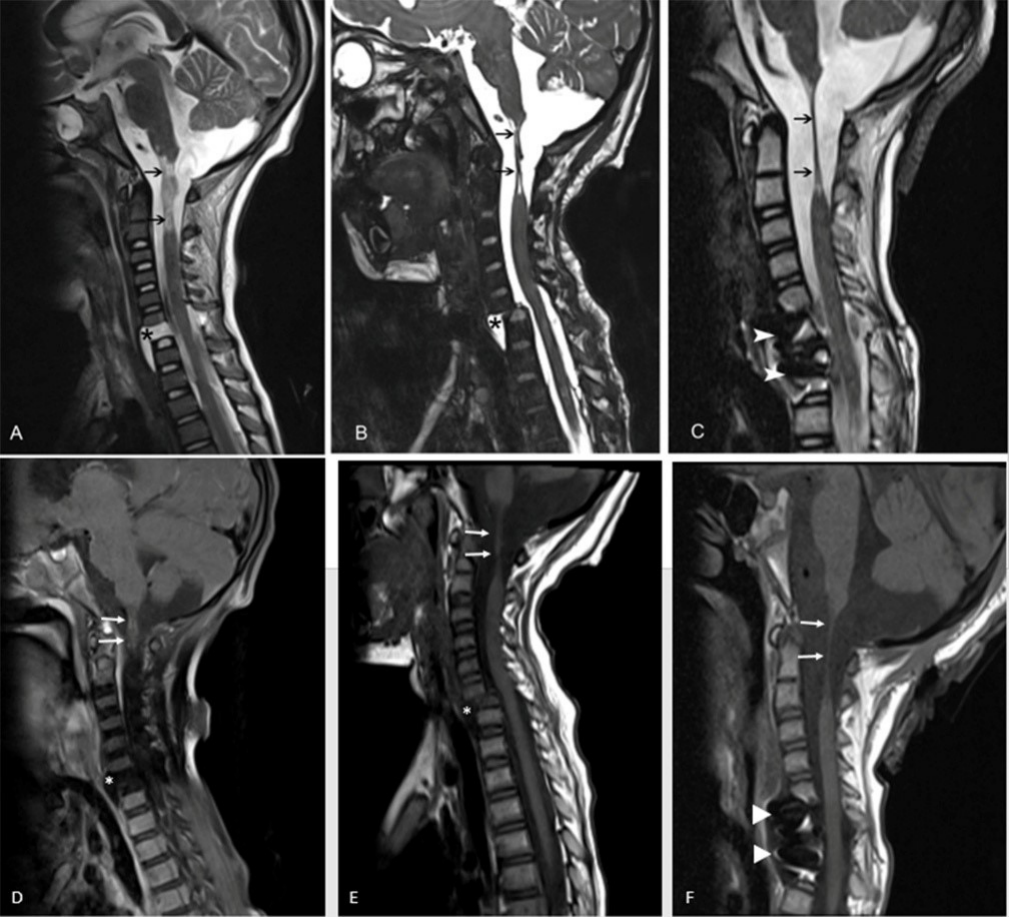
Magnetic resonance imaging at the third week following the accident **(A)** showing increased intensity at the junction of the medulla spinalis and medulla oblongata on T2-weighted images (black arrows). There is significant separation at the junction (black arrows). In addition, the separation of the C6-7 vertebral bodies and pseudomeningocele sac are seen (black asterisk). Sagittal T2-weighted magnetic resonance image taken at the second month after the accident **(B)** showing significant volume loss at the junction of the medulla spinalis due to axonal damage (black arrows). Spondylolisthesis developed at the level of the C6-7 vertebrae (black asterisk). Sagittal T2-weighted magnetic resonance image taken one year after the accident **(C)** showing the total volume loss of the medulla spinalis (black arrows). Surgical materials related to the operation were observed in the vertebral bodies (white arrowheads). Magnetic resonance imaging at the third week following the accident **(D)** showing decreased intensity at the junction of the medulla spinalis and medulla oblongata on contrast-enhanced sagittal T1-weighted images (white arrows). There is significant separation at the junction (white arrows). In addition, the separation of the C6-7 vertebral bodies and a pseudomeningocele sac are observed. There is no contrast enhancement in the pseudomeningocele sac (white asterisk). Sagittal T1-weighted magnetic resonance image taken at the second month after the accident **(E)** showing significant volume loss at the junction of the medulla spinalis due to axonal damage (white arrows). Spondylolisthesis developed at the level of the C6-7 vertebrae (white asterisk). Sagittal T1-weighted magnetic resonance image taken one year after the accident **(F)** showing the total volume loss of the medulla spinalis (white arrows). Surgical materials related to the operation are observed in the vertebral bodies (white arrowheads).

 A four-year-old boy who had been involved in a motor vehicle accident was admitted to our pediatric intensive care unit (PICU). During the accident, he was sitting in the child seat in the back of the car, with his seatbelt fastened. The accident occurred when the car was hit by another car when waiting at a red light. Cardiopulmonary resuscitation was applied to the boy for 10 minutes by the rescuers at the site of the accident. In the PICU, he was intubated carefully and mechanically ventilated, and all the other required medical treatments were administered. He was treated in the PICU for a long time. He was discharged with quadriplegia, tracheostomy, and home mechanical ventilator. During the follow-up period, consecutive magnetic resonance imaging (MRI) showed significant and rare findings (Figure 1).

 Whiplash injury, most commonly caused by motor vehicle accidents, has been defined as an acceleration-deceleration injury to the cervical spine.^1^ The abnormal S-shaped movement pattern of the lower cervical spine leads to injury.^2^ Its incidence is about 4 per 1000 persons.^3^ Affected patients often present with head, neck and upper thoracic pain and loss of motion. They may have cervical muscle spasm often without neurologic signs and symptoms.^4^ Although these patients often suffer from pain and loss of motion, life threatening conditions can also be seen. Computed tomography (CT) and MRI scans are generally required for patients with neurologic deficits or suspected damage to the spine.^5^ Here, we present the MRI scans of a child who suffered from severe whiplash injury. There was serious damage to his cervical spine and further complications were observed. The first important sign was significant separation at the junction of the medulla spinalis and medulla oblongata. The second and third signs were observed as a pseudomeningocele sac and spondylolisthesis development. All these findings are illustrated in detail in the consecutive MRI images shown in Figure 1. It is known that traumatic anterior cervical pseudomeningoceles are extremely rare.^6^ It is remarkable that these signs were seen in a four-year-old child and that the MRI scans are very illustrative.
